# Efficacy of Glyceryl trinitrate (GTN) to facilitate the rewarming process during cardiopulmonary bypass

**DOI:** 10.1186/s13019-020-01258-0

**Published:** 2020-08-10

**Authors:** Darren Mullane, Martin Lenihan, Ciara Hanley, Tom Wall, Irmina Bukowska, Michael Griffin, Georgina Flood

**Affiliations:** Mater Misercordiae University Hospital Division of Anesthesia, 7 Eccles Street, Dublin, Ireland

**Keywords:** Glyceryl trinitrate, Cardiopulmonary bypass, Rewarming, Temperature gradient, Lactate

## Abstract

**Background:**

Does Glyceryl trinitrate (GTN) administered during rewarming on cardiopulmonary bypass (CPB) impact: time to completion of rewarming prior to separation from CPB circuit, early post-op patient peripheral – core temperature gradient, time to maintenance of normothermia (core temperature > 36.5 °C) for minimum of 2 h in the initial post-op period, and plasma lactate concentrations initially post-CPB.

**Methods:**

Single centre prospective randomized trial conducted in the Mater Misericordiae University teaching hospital in Dublin Ireland. Trial registration: ISRCTN registry, ISRCTN10480871, registered 16th of August 2017. 82 patients enrolled. Patients randomised to low dose GTN infusion (0.01 mcg/kg/min) or higher dose GTN infusion (0.5 mcg/kg/min) during rewarming on CPB.

**Measurements and Main results:**

There was no significant difference between the treatment arms for the total time to being rewarmed, U = 759.0, *p* = 0.84. There were also no differences between the treatment arms for the time to achieve core temperature greater than 36.5 after two hours, U = 714.0, *p* = 0.52, the time to achieve plateau core skin temperature, U = 688.0, *p* = 0.37, and the post-intervention protamine lactate, U = 721.0, *p* = 0.56.

**Conclusions:**

Higher dose GTN infusion during rewarming on CPB does not improve peripheral-core temperature gradient post operatively and has no effect on post-operative lactate concentrations.

## Introduction

Cardiopulmonary bypass (CPB) has been the cornerstone for the evolution of cardiac surgery with its first regular use dating back to the 1950’s [1, 2]. The CPB machine facilitates surgery, allowing a still and bloodless field, whilst maintaining appropriate cardiac output, blood pressure and pulmonary functions, albeit not without its associated complications [3–5].

CPB results in vasoconstriction & reduction in blood flow to the peripheries which is further exacerbated by cooling [6, 7]. If rewarming back to normothermia is not complete at separation from CPB, then resultant hypothermia is seen with an increase in lactate levels following separation from the CPB circuit [8, 9].

Pharmacological vasodilation during rewarming is one way to counteract the potential deleterious effects of tissue hypoxia and impaired glucose metabolism, which occur as a result of peripheral vasoconstriction.

Deakin et al. showed that sodium nitroprusside during rewarming whilst on CPB, helped reduce the afterdrop phenomenon post operatively in the intensive care unit (ICU) [10]. Notably, time to extubation was also significantly reduced in this cohort of patients who received vasodilator therapy on rewarming. Glyceryl trinitrate (GTN) is another vasodilatory drug that has been used in the medical setting for over 100 years, for the cardiovascular benefits of coronary artery vasodilation and blood pressure optimization both in an outpatient and intra-operative setting [11, 12]. Ying-Hsuan Tai’s retrospective study in 2016, highlighted the benefit of GTN in attenuating the hyperglycemic response to cardiac surgery, whilst also ameliorating lactate levels in the intensive care setting [13]. GTN administration during rewarming, however, did not improve time to extubation, ICU length of stay, or hospital length of stay.

In our institution to date, we have been using GTN during rewarming on CPB but have found our exact dosing protocol to be quite variable. We therefore performed a small prospective observational study of GTN use during rewarming in our hospital in 2016. This showed evidence that an infusion at 0.5mcg/kg/min caused a slower rewarming process, but a more sustained normothermia post-operatively, with lower lactate levels in the initial post-operative period when compared to those with no infusion. On an extensive literature review however, we did not find any prospective large trials that assessed the efficacy of a higher dose GTN infusion when compared to a lower dose infusion during rewarming. The authors hypothesized that a higher dose GTN infusion during rewarming, would ameliorate temperature gradients and lactate levels in the acute post-operative period when compared to the significantly lower comparison.

## Methods

This single center prospective randomized trial was conducted in the Mater Misericordiae University Hospital Dublin from January 2017–December 2018. Our study was approved by the Mater Hospital Research & Ethics Committee (Ref 1/378/1869) and the trial was registered with the ISRCTN registry: ISRCTN10480871, EudraCT Number 2017–002785-44. Registered retrospectively.

All adult patients with capacity to consent undergoing cardiac surgery necessitating CPB were recruited for this trial. Exclusion criteria included age < 18 years old, allergy to GTN, cardiac surgery requiring deep hypothermic circulatory arrest, cardiac surgery not involving CPB (e.g. off-pump cardiopulmonary bypass grafting), lack of capacity to consent, and use of total intravenous anesthesia (TIVA) whilst on CPB. TIVA with propofol was excluded due to its potential beneficial vasodilating properties during CPB [14]. Based on our previous study we calculated our sample size to need over 59 patients to have a power value of 90% [15]. 82 patients were enrolled in total to allow for patient dropout.

The primary outcomes that we analyzed evaluated: if GTN administered during rewarming on CPB impacted (1) time to completion of rewarming prior to separation from CPB circuit, (2) early post-op patient peripheral-core temperature gradient, (3) time to maintenance of normothermia (core temperature > 36.5 °C) for minimum of 2 h in the initial post-op period (and including skin temperature reaching a plateau), and (4) plasma lactate concentrations initially post CPB.

Once written consent for the study was obtained by one of the trial authors, each study subject was randomized via a computer randomization programme to either a low or high dose GTN infusion rate during rewarming on CPB. The primary anaesthesiologist for each case was informed on which GTN dosing protocol to use on rewarming.

### Pre-operative assessment

Baseline demographics recorded included patient age at surgery, patient height, weight & Body Mass Index (BMI), planned surgery, left ventricular ejection fraction, Euroscore 2, patient co-morbidities & use of a vasodilator pre-operatively. Operating room temperature & patient temperature pre-operatively were also recorded.

### Intra operative assessment

Standard ASA (American Society of Anesthesiologists) monitoring at induction was used including: temperature measurement with core (bladder), peripheral (nasopharyngeal), & skin (temporal) monitors, invasive blood pressure measurement via an invasive arterial cannula, arterial blood gas monitoring (ABG) (standard measurements pre and post-CPB), invasive central venous pressure (CVP) monitoring, continuous end tidal carbon dioxide and inhalational anesthetic gas measurement, and transesophageal monitoring if indicated by procedure. Induction was at the discretion of the anaesthetizing consultant which included fentanyl, propofol, midazolam and an aminosteroidal muscle relaxant. Maintenance of anesthesia was conducted with a combination of volatile anesthetic and a fentanyl infusion.

### Commencement of CPB

Anesthesia was maintained by volatile anesthetic (sevoflurane) at 1.5% into the CPB circuit for both patient groups. Benzodiazepine and paralysis administration were at the discretion of the consultant anesthesiologist. Heparin dosing was used to keep ACT (activated clotting time) > 450 s. Mean arterial pressure (MAP) was maintained at a MAP of 60–80 mmHg with the aid of metaraminol and sevoflurane, with pump flow rates of 2.2 to 2.8 l/min/m2. Mild hypothermia was induced with cooling to 34 degrees as per surgical protocol. ABG monitoring was achieved every 30 min as per standard monitoring.

### Intervention

At initiation of rewarming, the patient received either: (A) High dose infusion: GTN infusion rate at 0.5 mcg/kg/min via CVP line or (B) Low dose infusion: GTN infusion rate at 0.01 mcg/kg/min via CVP line. Once core temperature and peripheral temperatures were > 36 °C and patient was ready for separation from CPB circuit, GTN infusion rate was set according to preference of the anesthesiologist and based on patient hemodynamics and co-morbidities. Recorded measurements included time taken for completion of rewarming process (both core and peripheral temperatures > 36 °C), total dose of GTN infusion (low or high rate), patient temperatures at commencement of CPB including core (bladder), peripheral (nasopharyngeal) & skin, lowest temperatures on CPB, and temperatures prior to separation from CPB.

### Post-operatively

Infusions of all vasoactive drugs were set according to clinical need and at the discretion of the anesthesiologist and / or intensivist as indicated for hemodynamic control.

Data recorded included doses of all bolus drugs administered, total doses of all drug infusions administered at application of dressings, dose of GTN administered during rewarming, total dose of metaraminol administered on CPB, and total dose of GTN administered in ICU in first 24 h post-operatively. Total Bypass time, cross clamp time & total surgical time were also recorded.

Temperature measurements post-operatively included temperature on application of surgical dressing (signifies end of surgery), ICU room temperature at arrival, hourly temperatures in ICU and lowest temperature recorded in ICU prior to extubation.

Lactate concentrations recorded perioperatively included: baseline ABG in OR, post-protamine ABG, initial ABG on arrival in ICU, and hourly ABG’s for the 1st 6 h in ICU. Lastly, time to extubation was also recorded for both groups.

### Statistical analysis

The statistical analysis was conducted using an “intention-to-treat” approach. The statistical assumption of normality for continuous distributions was assessed using Kolmogorov-Smirnov tests. Non-parametric Mann-Whitney U tests were performed to compare the treatment arms (Low GTN vs. High GTN) on the primary and secondary outcomes. Medians and interquartile ranges were reported and interpreted for each non-parametric comparison. Statistical significance was assumed at an alpha value of 0.05 and all statistical analyses were performed using SPSS Version 25 (Armonk, NY: IBM Corp.)

## Results

**Fig. 1:**
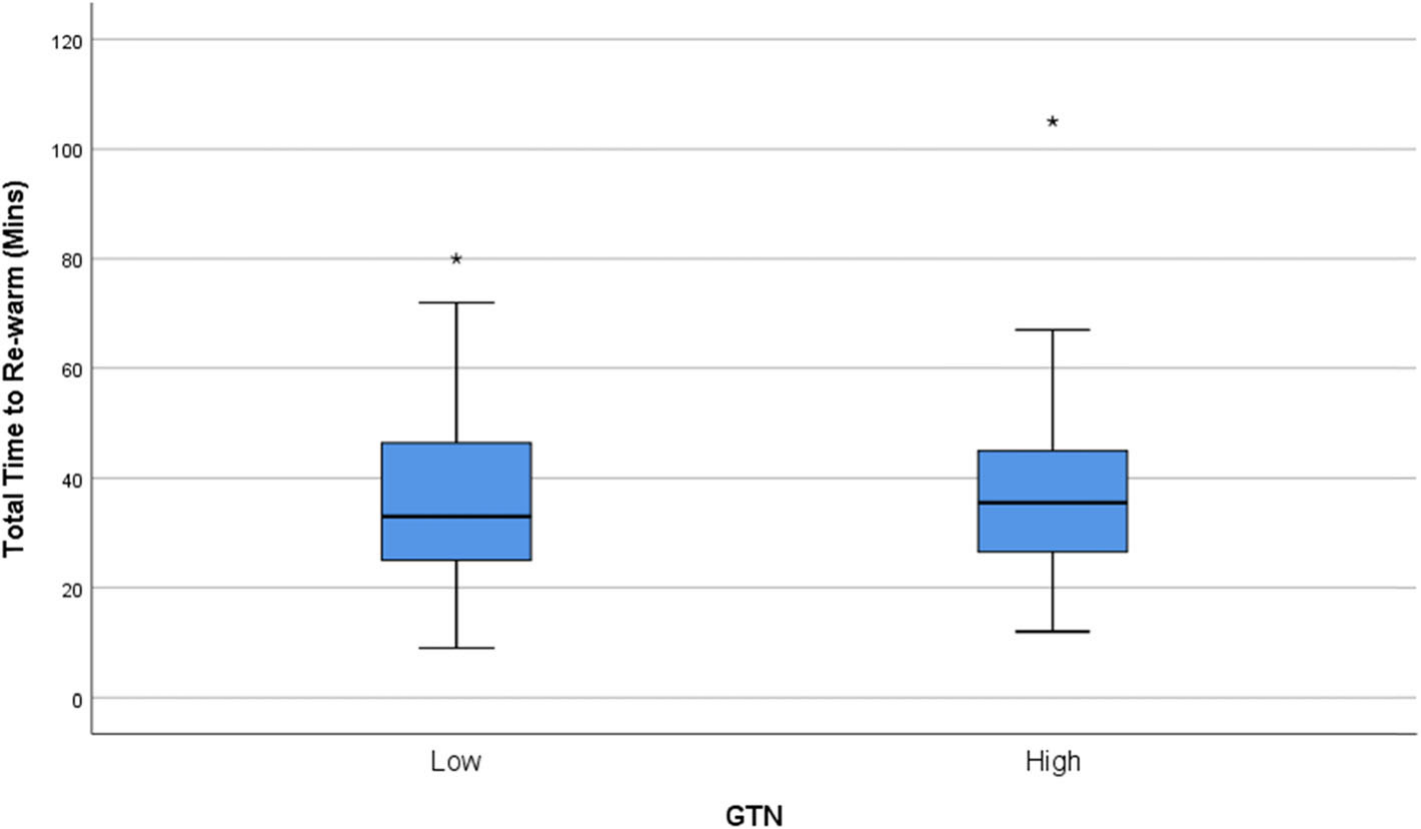
Box-plot for Total Time to Re-warm

**Fig. 2:**
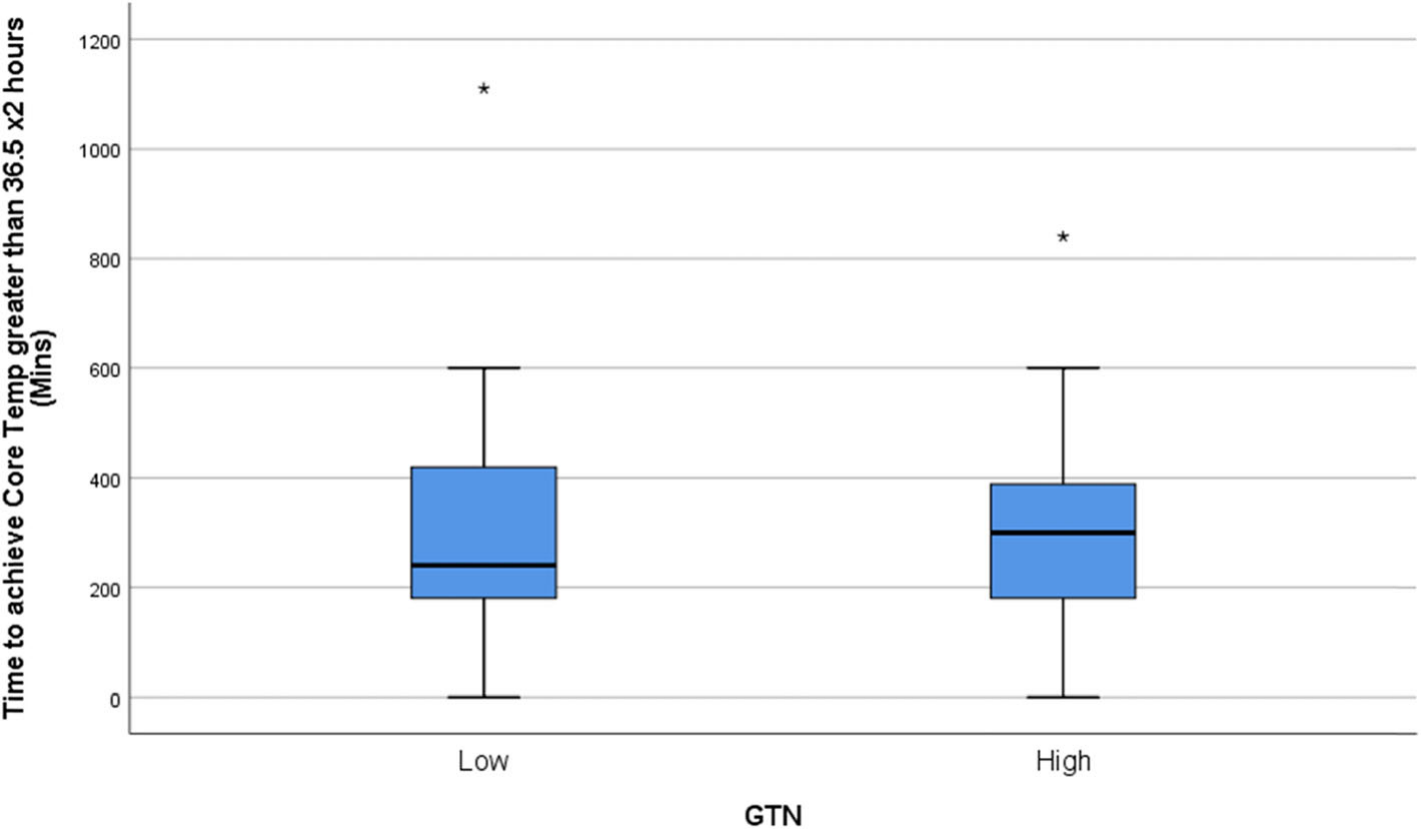
Box-plot for Time to Achieve Core Temperature Greater Than 36.5 × 2 Hours

**Fig. 3:**
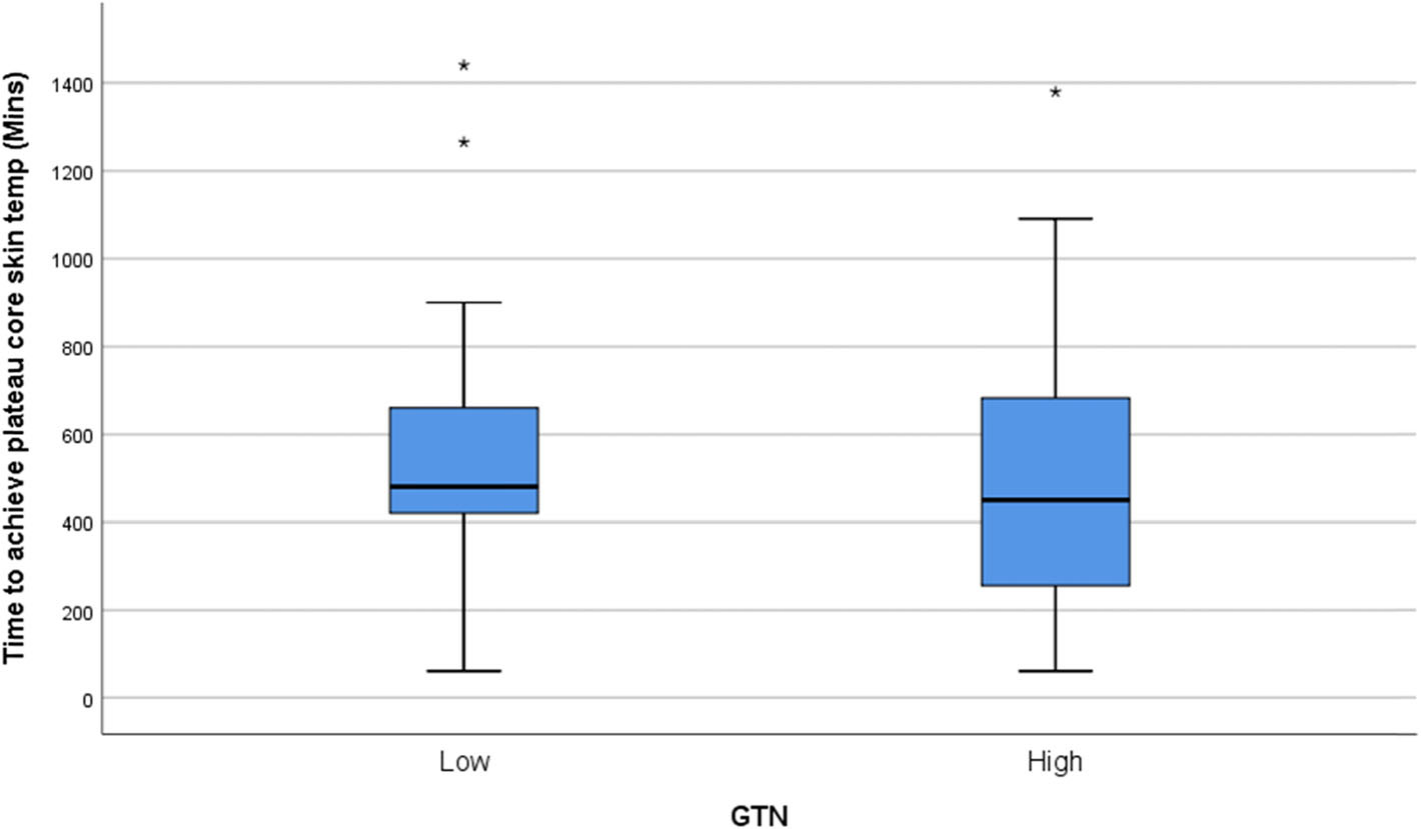
Box-plot for Time to Achieve Plateau Core Skin Temperature

**Fig. 4:**
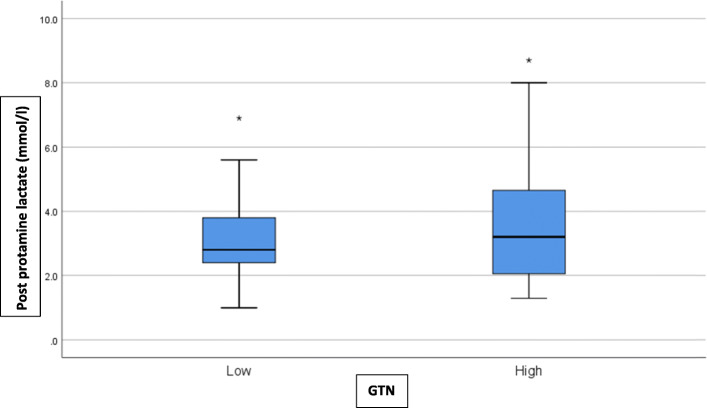
Box-plot for Post-Protamine Lactate

**Fig. 5:**
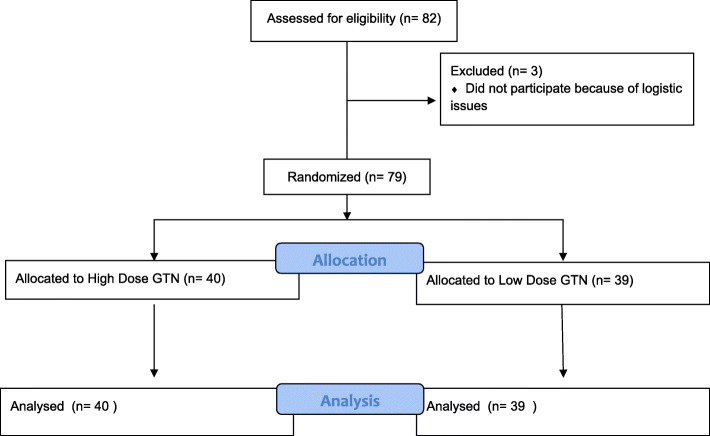
CONSORT flow diagram reflecting recruitment of patients [23]

Kolmogorov-Smirnov tests found that all continuous outcome distributions violated the statistical assumption of normality. Non-parametric Mann-Whitney U tests were used for treatment arm comparisons of each outcome of interest. Medians and interquartile ranges for the non-parametric comparisons are presented in Table 1 and depicted visually using box-plots in Figs. 1, 2, 3, and 4. Figure 5 outlines the allocation and randomisation of patients during the study in the CONSORT flow diagram format [16]. There was no significant difference between the treatment arms for the total time to being rewarmed, U = 759.0, *p* = 0.84 as presented in Table 1 and depicted in Fig. 1. Table 1 also highlights that there were no differences between the treatment arms for the time to achieve core temperature greater than 36.5 after two hours, U = 714.0, *p* = 0.52, the time to achieve plateau core skin temperature, U = 688.0, *p* = 0.37, and the post-intervention protamine lactate, U = 721.0, *p* = 0.56.

**Table 1:** Comparison of GTN Treatment Arms of Primary and Secondary Outcomes

Outcome	Low GTN	High GTN	*p*-value
Total time to rewarming (minutes)	33.0 (23.0)	35.5 (19.0)	0.84
Time to achieve core temperature greater than 36.5 after two hours (minutes)	240.0 (240.0)	300.0 (210.0)	0.52
Time to achieve plateau core skin temperature (minutes)	480.0 (240.0)	450.0 (438.0)	0.37
Post-intervention Protamine Lactate (mmol/l)	2.8 (1.4)	3.2 (2.7)	0.56

Note: values are median (interquartile range)

Table 2 highlights the differences between both research groups. Mean age was 61.5 years in both groups. There was no significant difference observed in Euroscore 2 scores, BMI, total bypass time, total cross clamp time, and duration of surgery.

**Table 2:** Differences Between GTN Groups

Variable	Low GTN	High GTN	*p*-value
Age*	61.5 (11.9)	61.5 (6.96)	0.99
BMI*	28.8 (4.9)	28.4 (4.9)	0.77
Cross Clamp Time (mins)*	102.9 (32.1)	100.4 (32.0)	0.73
Euroscore 2**	1.6 (1.0)	1.8 (2.0)	0.72
Total Bypass Time (mins)**	130.0 (61.0)	123.5 (47.0)	0.64
Total Time to Re-warm (mins)**	33.0 (23.0)	35.5 (19.0)	0.84
Total Dose of GTN During Re-warming (mcg)	25.0 (25.0)	1506.0 (856.0)	< 0.001
Duration of Surgery (mins)**	312.0 (120.0)	315.5 (65.0)	0.40
Time to Extubation (mins)**	900.0 (400.0)	900.0 (360.0)	0.99
ICU Arrival Lactate (mmol/l)**	2.3 (1.9)	1.8 (2.6)	0.97
First Hour Lactate (mmol/l)**	1.8 (1.5)	1.7 (2.6)	0.94
Second Hour Lactate (mmol/l)**	2.0 (1.7)	2.0 (2.6)	0.98
Third Hour Lactate (mmol/l)**	1.9 (1.7)	1.8 (1.9)	0.97
Fourth Hour Lactate (mmol/l)**	2.1 (2.2)	2.1 (2.2)	0.67
Fifth Hour Lactate (mmol/l)**	2.1 (2.9)	2.0 (1.9)	0.74
Sixth Hour Lactate (mmol/l)**	2.2 (2.3)	2.6 (2.6)	0.87

Note: * Values are mean (standard deviation), ** Values are median (interquartile range)

## Discussion

This prospective study is the first research trial to evaluate a higher dose GTN infusion vs lower dose GTN infusion during rewarming, to ameliorate the deleterious effects of CPB in Cardiac Surgery. We recruited over 80 patients to have our study adequately powered, but we found no statistically significant differences between any of our primary endpoints when comparing both dosing protocols. Time to achieve plateau temperature between core and skin was equal in both groups, as was time to achieve core temperature > 36.5. This may account for the lack of statistically significant differences between the initial post-operative lactates. There was also no statistical difference found between the hourly post-operative lactates with either group in the ICU setting, despite the well know association of prolonged CPB time with elevated lactates post operatively [17–22]. Baseline demographic data, total CPB time, total time to rewarm & total surgery time were comparable between both groups including Euroscore 2. This may also explain the comparable time to extubation found between both groups in the setting of a matching post-operative lactate trend. Our results did not replicate the previous positive findings from our earlier study or Ying-Hsuans retrospective review [14, 15].

The mean dose of GTN administered in the low dose group was 25 micrograms compared to 1506 micrograms in the higher dose GTN group. The authors felt this overall minimal dose was unlikely to contribute to any significant vasodilator effect, however it cannot be fully excluded.

One limitation of our study relates to the notable side effects of GTN. Venodilation with GTN has been associated with a higher fluid requirement during surgery to maintain intravascular volume and cardiac output [13, 23]. We did not however, look at morbidity in the ICU setting outside of early lactate trend and time to extubation. Time to extubation between both groups was comparable, but respiratory complications post operatively were not recorded.

One interesting study that showed promise by Kumar et al., looked at propofol vs GTN on the efficacy of rewarming along with extra volume added during CPB [14]. This study showed a benefit of propofol over GTN in reducing the afterdrop phenomenon, albeit the numbers were much smaller with only 10 patients in each arm. This is one area that may have potential if investigated on a larger scale.

## Conclusion

The authors demonstrated in this study that a higher dose GTN infusion during rewarming on CPB does not improve peripheral-core temperature gradient post operatively, and has no effect on post-operative lactate concentrations. Time to extubation was also unaffected.

## Data Availability

All data generated or analysed during this study are included in this published article [and its supplementary information files.

## Abbreviations

GTN: Glyceryl trinitrate
CPB: Cardiopulmonary Bypass
ICU: Intensive Care Unit
TIVA: Total Intravenous Anesthesia
BMI: Body Mass Index
ASA: American Society of Anesthesiologists
ABG: Arterial Blood Gas
CVP: Central Venous Pressure
ACT: Activated Clotting Time
MAP: Mean Arterial Pressure
